# Alcohol and Gut-Derived Inflammation

**DOI:** 10.35946/arcr.v38.2.02

**Published:** 2017

**Authors:** Faraz Bishehsari, Emmeline Magno, Garth Swanson, Vishal Desai, Robin M. Voigt, Christopher B. Forsyth, Ali Keshavarzian

**Affiliations:** Faraz Bishehsari, M.D., Ph.D., is an Assistant Professor; Garth Swanson, M.D., is an Assistant Professor; Vishal Desai, M.D., is a Physician; Robin M. Voigt, Ph.D., is an Assistant Professor; Christopher B. Forsyth, Ph.D., is an Associate Professor; and Ali Keshavarzian, M.D., is a Professor, all in the Department of Internal Medicine, Division of Gastroenterology, Rush University Medical Center, Chicago, Illinois. Emmeline Magno, M.D., is an Internist in the Department of Internal Medicine, Rush University Medical Center, Chicago, Illinois

**Keywords:** Alcohol consumption, alcohol use, abuse, and dependence, chronic alcohol use, organ damage, gastrointestinal (GI) tract, liver, metabolites, gut-derived inflammation, intestinal inflammation, intestinal microbiota

## Abstract

In large amounts, alcohol and its metabolites can overwhelm the gastrointestinal tract (GI) and liver and lead to damage both within the GI and in other organs. Specifically, alcohol and its metabolites promote intestinal inflammation through multiple pathways. That inflammatory response, in turn, exacerbates alcohol-induced organ damage, creating a vicious cycle and leading to additional deleterious effects of alcohol both locally and systemically. This review summarizes the mechanisms by which chronic alcohol intake leads to intestinal inflammation, including altering intestinal microbiota composition and function, increasing the permeability of the intestinal lining, and affecting the intestinal immune homeostasis. Understanding the mechanisms of alcohol-induced intestinal inflammation can aid in the discovery of therapeutic approaches to mitigate alcohol-induced organ dysfunctions.

The gastrointestinal (GI) tract, as the first line of contact with anything ingested into the body, is at particular risk for damage by toxins. And mounting research suggests that poor gastrointestinal health plays a significant role in the body’s overall health. Connecting the dots, anything that may cause GI damage, may have consequences far beyond the intestines. In fact, researchers have begun to discover that alcohol, particularly if consumed chronically and in larger amounts, induces a process initiated in the gut that promotes inflammation throughout the body (Patel et al. 2015). This alcohol-induced intestinal inflammation may be at the root of multiple organ dysfunctions and chronic disorders associated with alcohol consumption, including chronic liver disease, neurological disease, GI cancers, and inflammatory bowel syndrome. This review summarizes the mechanisms by which chronic alcohol intake leads to intestinal inflammation. These mechanisms include alcohol’s influences on intestinal microbiota, on the integrity of the barrier between the intestine and the rest of the body, and on immune function within and outside the GI tract. The factors that can modify alcohol-induced gut inflammation and organ damage and the resulting pathologies that are a consequence of gut-derived inflammation are described. Although there may be large gender, racial, and interindividual variations in alcohol’s effect on the GI tract, depending on differences in alcohol absorption, distribution, and elimination, they are not the focus of the current review.

## Alcohol Metabolism and the Gut

Once consumed, alcohol is absorbed mainly in the upper intestinal tract by diffusion and then enters the liver via the portal vein. Therefore, the effect of alcohol on the distal small intestine and colon should largely come from its circulatory levels. That said, the luminal concentration of alcohol in the latter parts of the small intestine, close to the colon, reaches up to 200 mg/100 ml within an hour of drinking 2 to 2 1/2 standard alcoholic drinks (0.8 g/kg) (Halsted et al. 1973).

The majority of alcohol metabolism in humans occurs in the liver, in cells called hepatocytes. During social drinking, defined here as an average of two standard drinks, the body typically processes the ingested alcohol with no deleterious effects through a process called oxidative conversion, during which the enzyme alcohol dehydrogenase (ADH) converts alcohol into the toxin acetaldehyde. Acetaldehyde dehydrogenase (ALDH) then converts acetaldehyde into acetate. Another alcohol metabolism pathway, the microsomal ethanol–oxidizing system (MEOS), handles a small portion of alcohol metabolism in social drinkers but a significant portion of alcohol metabolism when the body needs to process larger amounts of alcohol. MEOS leads to the production of oxygen free radicals, which can cause cellular damage. Although the majority of alcohol metabolism occurs in hepatocytes, the enzymes involved in the oxidative metabolism of alcohol also are present in the intestinal mucosa (Cederbaum 2012) and intestinal bacteria also produce acetaldehyde in the GI tract. In addition, less commonly, nonoxidative alcohol metabolism occurs in the intestines via reactions with membrane phospholipids and/or free fatty acids. This alternative pathway may become particularly relevant when intestinal injuries occur after chronic alcohol consumption (Elamin et al. 2013*a*).

Therefore, both the small and large intestine can be affected by alcohol and its metabolites as the result of its oxidative and nonoxidative metabolism. Metabolism of alcohol in the GI tract can then lead to disruption of tissue homeostasis toward a chronic state of intestinal inflammation (Patel et al. 2015; Rao et al. 2004). As will be discussed in this review, mounting evidence shows that alcohol induces intestinal inflammation through various pathways, including changes in intestinal microbiota composition (Engen et al. 2015; Mutlu et al. 2012) and function (Couch et al. 2015), increased permeability of the intestinal mucosa (Tang et al. 2015), and disruptions of the immune system of the intestinal mucosa (Cook 1998).

## Underlying Mechanisms for Alcohol and Gut-Derived Inflammation

### Alcohol and Intestinal Microbiota

The intestine houses more than 500 bacterial species and achieves bacterial homeostasis when the ratio between “good” bacteria and pathogenic bacteria is appropriately balanced. “Dysbiosis” occurs when disease or environmental factors disrupt the bacterial balance (Belizário and Napolitano 2015). Disruption to the normal gut flora also occurs when there is an overall overgrowth of bacteria. Studies show that alcohol promotes both dysbiosis and bacterial overgrowth (Mutlu et al. 2012; Schnabl and Brenner 2014), which in turn leads to an increase in the release of endotoxins, produced by gram-negative bacteria (Rao et al. 2004). Endotoxins activate proteins and immune cells that promote inflammation (Elamin et al. 2013*a*; Keshavarzian et al. 2009). This section discusses evidence supporting alcohol’s role in altering intestinal microbiota.

### Bacterial Overgrowth

Studies in animals and humans confirm that alcohol increases intestinal bacteria (Canesso et al. 2014). This overgrowth may be stimulated directly by alcohol, but some studies suggest that it also could be an indirect byproduct of poor digestive and intestinal function caused by alcohol consumption. For example, studies of patients with liver cirrhosis (both caused by alcohol and not) found an association between patients with abnormal intestinal motility—the intestine’s ability to move food along— and bacterial overgrowth (Chang et al. 1998). Other studies found a connection between alcohol, bile acid, and bacterial overgrowth. Specifically, alcohol can alter bile-acid metabolism and, in turn, bile acids can affect intestinal bacteria (Schnabl and Brenner 2014). Studies in rats show that alcohol decreases certain bile acids (Xie et al. 2013) and treating rats with bile acids reversed bacterial overgrowth (Lorenzo-Zúñiga et al. 2003).

### Bacterial Dysbiosis

More recent studies use DNA sequencing technology to assess intestinal microbiota populations and indicate a correlation between alcohol and changes in the ratio between beneficial or “good” bacteria, such as strains of *Lactobacillus* and *Bifidobacterium*, and pathogenic bacteria, such as proteobacteria and bacilli (Canesso et al. 2014). For example, mice chronically fed alcohol display a decrease in good bacteria and an increase in bacteria that boost endotoxin production (Bull-Otterson et al. 2013). In another study, researchers found a significant shift in intestinal microbiota composition in rats chronically fed alcohol, but they could prevent the shift by giving the rats *Lactobacillus GG* bacteria and a diet that included probiotic oats (Mutlu et al. 2009). Connecting dysbiosis to alcohol-induced health problems, several studies find that probiotic and synbiotic interventions, which stimulate the growth of beneficial bacteria, attenuate liver injury in rats (Forsyth et al. 2009) and liver dysfunction in cirrhotic patients (Liu et al. 2004). Alcohol-induced bacterial overgrowth also may increase the risk of inflammation because intestinal bacteria can independently metabolize alcohol, producing excess acetaldehyde in the colon, which increases production of proinflammatory alcohol metabolites (Zhong and Zhou 2014).

### Alcohol-Induced Intestinal Hyperpermeability

The intestinal barrier regulates the passage of materials between the GI tract and the bloodstream, allowing for the absorption by the blood of key nutrients and preventing the absorption of noxious substances. It is made up of a layer of water, mucous gel, and epithelial and connective tissue. The epithelial layer can become leaky or “permeable,” allowing pathogens and other deleterious substances into the bloodstream.

Studies in humans demonstrate that a subset of people with alcohol use disorder (AUD) in fact have increased intestinal permeability, measured using a method called Cr-EDTA, which examines excretion of orally administered chromium (Leclercq et al. 2014). In addition, those people with AUD and with increased permeability are more likely to have liver disease (Keshavarzian et al. 1999), indicating that intestinal permeability may be a mediator of organ damage in some people with AUD. Another study showed that not only is gut permeability increased in people with AUD, it is increased enough to allow large macromolecules through the intestinal barrier (Parlesak et al. 2000). Endotoxins— also known as lipopolysaccharides (LPS)—are large macromolecules and, as expected, the same study found that plasma endotoxin levels increased in parallel with increases in gut permeability (Parlesak et al. 2000).

But exactly how does alcohol induce intestinal permeability? The short answer is by disrupting the epithelial cells themselves (transepithelial permeability) and by disrupting the spaces between the epithelial cells (paracellular permeability), which consist of tight junctions, the cytoskeleton, and several associated proteins (see figure below). Trans-epithelial permeability is caused by direct cellular damage. For example:

- Alcohol causes cell death (Pijls et al. 2013), which leads to changes in the intestine that include mucosal ulcerations, erosions, and loss of epithelium mainly at the villi tips (Rocco et al. 2014);
- Acetaldehyde forms DNA adducts that cause direct cellular damage (Malaguarnera et al. 2014); and
- Reactive oxygen species (ROS) released during alcohol metabolism cause direct cellular damage via oxidative stress (Forsyth et al. 2014).

Alcohol and its metabolites cause paracellular permeability by acting on the tight-junction complex, which melds two adjoining cells together. For example, acetaldehyde destabilizes tight junctions by redistributing proteins (Dunagan et al. 2012); alcohol and its metabolites alter the expression of tight-junction proteins (Wang et al. 2014); and alcohol nonoxidative metabolites cause tight-junction redistribution, disrupting its barrier function (Elamin et al. 2013*b*). In addition, studies show that alcohol destabilizes cells’ cytoskeletons, the cell borders that give them their structure (Elamin et al. 2014). There also is growing evidence that alcohol causes the overexpression of microRNAs (miRNAs), which are small stretches of noncoding RNA that silence gene expression (Tang et al. 2014). Specifically, alcohol can lead to overexpression of miRNAs that influence genes associated with gut-barrier integrity (Lippai et al. 2014).

### Alcohol Modulation of Mucosal Immunity

Gut inflammation results from an inflammatory response mounted by the immune system against alcohol and its metabolites. Alcohol affects intestinal mucosal immunity via several mechanisms (see sidebar). In particular, it may first decrease the innate immune response in the mucosa, resulting in increased susceptibility to intestinal pathogens (Zhou et al. 2013). Subsequently, as found in studies in cell cultures, alcohol may trigger an immune system response and upregulation of molecules that promote the inflammatory response, including a release of inflammatory immune cells, such as leukocytes and mast cells (Fleming et al. 2001).

As mentioned earlier, alcohol-related bacterial overgrowth and dysbiosis may lead to increased endotoxin production in the gut, which can bind to cells on the intestinal mucosa, causing local inflammation, and translocate to extraintestinal sites, causing systemic inflammation (Leclercq et al. 2014). Studies also show that alcohol can directly modulate both innate and adaptive immunity, further contributing to gut and gut-derived inflammation. For example, a study in mice found that alcohol inhibits the intestine’s immune response for clearing hazardous bacteria (Sibley and Jerrells 2000), and other studies find that alcohol suppresses intestinal mucosal immune cell activity (Cook 1998). Additional studies find myriad ways that alcohol affects mucosal immunity, including the following:

- By reducing the amount of anti-microbial molecules intestinal cells secrete, which leads to bacterial overgrowth (Schnabl and Brenner 2014);
- By suppressing the signaling molecule, interleukin-22, which negatively affects antimicrobial peptides (e.g., Reg3β and Reg3γ) and intestinal mucosal integrity (Rendon et al. 2013); and
- By suppressing signal molecules and immune T cells and thereby suppressing the intestinal mucosal immune response and bacterial clearance (Trevejo-Nunez et al. 2015).

## Modifying Factors for Alcohol-Induced Gut-Derived Inflammation

As described above, alcohol causes gut-derived inflammation, which is related to other alcohol-associated pathologies. However, not all people with AUD develop disease, and those who do have varying degrees of disease severity. Although the extent of disease depends in large part on the extent of alcohol use and likely involves inherent individual characteristics, including genetics, race, and age, there are some adjustable factors that affect alcohol-induced intestinal inflammation and, therefore, may prevent or slow the progression of alcohol-related disease. Here, we discuss the roles of two adjustable environmental factors: circadian rhythm and diet.

### Circadian Disruption

Circadian rhythm, also known as the biological clock, refers to an internal cycling of various biological processes. Chronic alcohol use can lead to a disrupted biological clock, which in turn can have a wide range of health-related consequences.

In terms of gut-related inflammation, studies in cell cultures, mice, and humans suggest that a disrupted circadian rhythm exacerbates alcohol-related gut leakiness. For example, one study (Summa et al. 2013) found that alcohol-fed CLOCK mutant mice—who have a disrupted circadian cycle—showed more evidence of gut leakiness than alcohol-fed wild-type mice. A study in humans (Swanson et al. 2015), including a group of shift workers who often have disrupted circadian rhythm, came to a similar conclusion. The researchers assessed circadian rhythm by measuring participants’ blood melatonin levels, using low melatonin as a marker for disrupted circadian rhythm. They found that low melatonin correlated with gut leakiness in people with AUD.

Although it is unclear how circadian disruption amplifies alcohol-induced gut permeability, there are some hints from recent studies. For example, gut microbes have circadian oscillations, and circadian disruption can lead to dysbiosis in mice fed a high-fat diet (Voigt et al. 2014), which in turn can induce intestinal inflammation and hyperpermeability. In addition, timing of lipid metabolism and bile-acid synthesis are regulated by the local hepatic circadian rhythm (Bailey et al. 2014). Together, the evidence on circadian rhythm suggests a looping cycle where circadian disruption promotes alcohol-induced intestinal inflammation and alcohol disturbs circadian rhythm, which may further propagate intestinal hyperpermeability and inflammation.

### Diet

Various studies show that nutrition can modify alcohol-induced gut inflammation and, subsequently, extraintestinal organ damage. Because people with AUD typically have altered diet composition, a focus on changing dietary habits might attenuate alcohol-related diseases. The following section reviews a sampling of studies on different diets and alcohol use.

**Alcohol’s Effect on Immunity and Inflammation**

Alcohol can induce intestinal inflammation through a cascade of mechanisms that subsequently lead to inflammation and organ dysfunction throughout the body, in particular in the liver and brain. One mechanism is by increasing bacterial loads and the permeability of the intestinal wall (see figure) allowing bacteria to leak through, leading to local and systemic effects by affecting mucosal immunity and via endotoxin release, respectively. Alcohol also affects mucosal immunity by suppressing one of the intestine’s main lines of defense against bacteria, Paneth cells that secrete antibacterial compounds. Suppressed Paneth cells secrete fewer antibacterial compounds, which can allow additional intestinal bacteria overgrowth and allow their byproducts (i.e., endotoxins) entrance through the intestinal barrier. The bacteria, via endotoxins, trigger an inflammatory response by the intestine’s immune system, causing a release of proinflammatory cytokines. The endotoxins and cytokines can then enter the liver, directly interacting with hepatocytes and with liver immune cells, causing local cytokine release that leads to fibrosis and causes additional inflammation. The gut inflammation can also spread endotoxins and cytokines into the bloodstream where they can enter the central nervous system (CNS), causing neuroinflammation.

**Figure:**
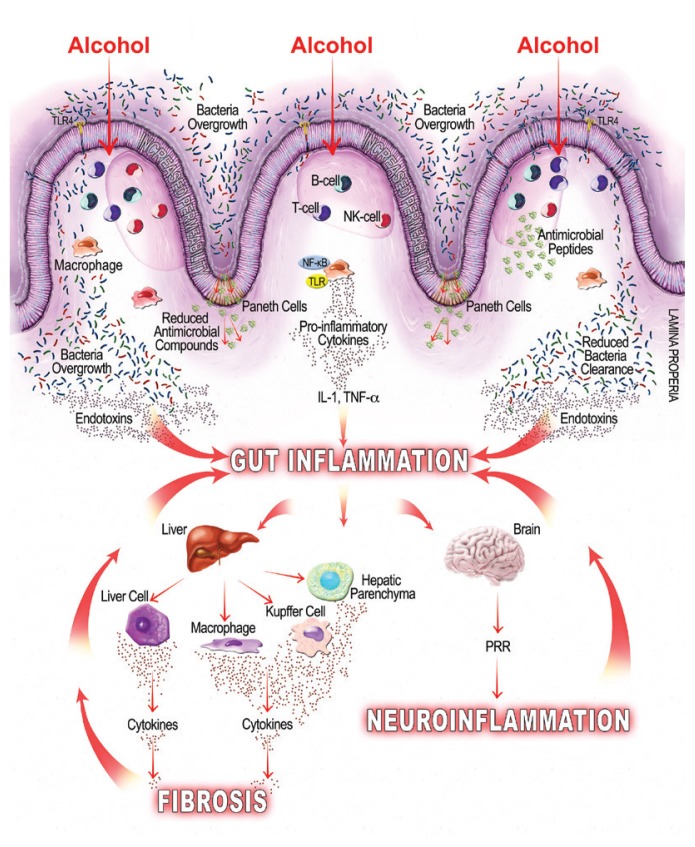

#### Fat

Studies examining high-fat diets find conflicting results. Some find fats propagate alcohol’s effects on the intestine, and some find they attenuate alcohol’s harmful effects. The contrast likely reflects the variety of fats found in high-fat foods. Generally, studies seem to support the idea that unsaturated fats increase gut permeability and some kinds of saturated fats are protective.

Studies have examined the effects of several types of saturated fats given as supplements to alcohol-exposed mice. One (Cresci et al. 2014) found that tributyrin, a triglyceride fat found in butter and margarine, prevented alcohol-induced tight-junction disruption, which in turn protects against intestinal hyperpermeability. Another (Chen et al. 2015*a*) examined saturated long-chain fatty acids (SLCFAs), which are found in coconut oil, peanut oil, and dairy products. The researchers observed that the intestinal bacteria in mice chronically fed ethanol produced far less SLCFAs than mice not fed ethanol, and they also had lower levels of tight-junction proteins. That changed after the researchers gave the ethanol-fed mice SLCFA supplementation. Indeed, the mice given supplementation had higher levels of tight-junction proteins than ethanol-fed mice without supplementation. SLCFA supplements also prevented dysbiosis (Chen et al. 2015*a*).

Unsaturated fats had less favorable effects. In one study (Kirpich et al. 2012), mice fed alcohol and unsaturated fats had increased fatty liver changes and suppressed mRNA expression of tight-junction proteins compared with mice fed alcohol and saturated fat. These findings suggest that an unsaturated-fat diet in conjunction with chronic alcohol use increases intestinal permeability.

#### Oats*.*

Oats, which are rich in fat, fiber, protein, vitamins, and minerals, have long been associated with cardiovascular health and, more recently, examined for a possible role in gastrointestinal health. Several preclinical studies suggest that oats may attenuate alcohol’s deleterious effects on the digestive system. In one study (Keshavarzian et al. 2001), two groups of rats received increasing doses of alcohol and either oats or regular rat chow for a period of 10 weeks. The oats-fed rats had significantly lower endotoxin levels than the chow-fed animals. Another study (Tang et al. 2009) found that alcohol-fed rats given oat supplementation showed fewer signs of gut inflammation and alcohol-induced hyperpermeability than rats fed alcohol and regular rat chow. More recently, researchers examined supplementation with glutamine, an amino acid found in oats. The study in mice found that glutamine supplements ameliorated alcohol-induced intestinal leakiness and improved alcohol-induced liver injury (Chaudhry et al. 2016).

#### Vitamins and Minerals

People with AUD often are deficient in certain vitamins and minerals, including zinc and vitamin D, either from direct effects of alcohol consumption or poor diet. Those deficiencies, in turn, may have deleterious effects on the digestive system. A study in mice (Zhong et al. 2013) found a relationship between zinc deficiency and gut leakiness. The study compared mice fed alcohol and a zinc-deficient diet with mice fed alcohol and a zinc-adequate diet. The zinc-deficient mice showed increased intestinal permeability and higher plasma endotoxin levels (for more on zinc, see the article by McClain).

Another study, conducted in intestinal cell culture and mice, examined whether vitamin D might protect gut health from alcohol exposure. In the cells, treating with vitamin D protected the cells from ethanol damage. In the mice, higher vitamin D levels measured in blood correlated with increased resistance to changes that lead to intestinal injury (Chen et al. 2015*b*). These findings suggest that vitamin D deficiency may promote the deleterious effects of alcohol on the gut barrier and, perhaps, that vitamin D supplementation may attenuate those effects.

## The Clinical Relevance of the Alcohol-Induced Gut-Derived Inflammation

Alcohol-induced gut inflammation is believed to promote several disease states both within the GI tract, in the form of gastrointestinal cancers and inflammatory bowel disease, and outside the GI tract, in the form of, for example, liver disease and neuroinflammation (Rao et al. 2004). The following section briefly reviews a sample of the conditions associated with alcohol-related gut inflammation.

### Alcohol and GI Cancers

Chronic alcohol consumption is associated with increased risk of major gastrointestinal cancers including cancer of the esophagus, stomach, and colon (colorectal cancer). The risk generally increases as alcohol consumption increases and in combination with other lifestyle-related factors, such as smoking tobacco or metabolic syndrome. And although alcohol was initially thought to act as a direct carcinogen, research instead suggests that alcohol-induced gut inflammation may be at fault (Thrift et al. 2011).

Systemic inflammation seen in metabolic syndrome and obesity increases risk of several types of epithelial cancers, including those in the gastrointestinal tract (Feakins 2016), suggesting that the systemic inflammatory state created by alcohol-induced gut inflammation also may contribute to alcohol-induced carcinogenesis in the GI tract and other organs. This process can snowball because, as cells transition to a cancerous state, ADH activity increases while ALDH activity may decrease (Testino et al. 2011). This leads to an increased oxidation rate and a decreased ability to clear alcohol metabolites (Testino et al. 2011), which in turn can further promote carcinogenesis through direct effects on DNA, oxidative stress, and gut inflammation (Jelski and Szmitkowski 2008).

## Alcohol and Inflammatory Bowel Disease (IBD)

Several lifestyle factors such as smoking and diet affect the incidence and severity of IBD, most likely by modulating gut inflammation (Swanson et al. 2010). Alcohol consumption also may influence the course of IBD through associated gut inflammation (McGuckin et al. 2009); however, its effect in patients with IBD only has been studied in a few small studies. One study (Swanson et al. 2011), for example, examined the impact of 1 week of moderate (24 g to 36 g ethanol daily) red wine consumption on clinical disease activity and other noninvasive markers associated with increased risk of future disease flare. The study found no significant changes in indices of clinical disease but did find subclinical increases in markers for disease activity, including intestinal permeability. Such findings suggest that chronic alcohol consumption could increase the long-term risk for disease flare in IBD and supports the need for additional study.

## Gut–Liver Axis

Approximately 20 to 30 percent of heavy drinkers (people who drink more than 30 grams/day for at least 10 years) develop clinically significant alcoholic liver disease, including alcoholic steatohepatitis and cirrhosis (Gramenzi et al. 2006). Several factors, such as the amount and duration of alcohol consumption, obesity, and gender, seem to moderate a person’s risk and progression of alcoholic liver disease. In addition, studies find that alcohol-induced gut inflammation can contribute to liver injury by increasing intestinal permeability and the likelihood that gut-derived endotoxins enter the liver. One study (Keshavarzian et al. 1999) found that people with AUD who also have liver disease are much more likely to have intestinal permeability: more than 40 times more likely than people without AUD and more than 20 times more likely than people with AUD who do not have liver disease. In alcohol-fed rats, gut leakiness is evident 2 weeks after alcohol initiation; after another 2 weeks, endotoxemia develops and then liver injury, suggesting an intermediary role for endotoxemia on liver injury (Keshavarzian et al. 2009).

Once gut leakiness begins, endotoxins can enter the liver via the portal vein that drains from the gut. In the liver, gut-derived substances interact with the liver’s hepatocytes, parenchymal cells, and immune cells. Alcohol exposure increases LPS levels in portal and systemic circulation (Wheeler et al. 2001), and that can have a host of deleterious effects:

- Initiating endotoxin-mediated hepatocellular damage by activating the innate immune system and leading to an increase in ROS and inflammatory cytokines, leukotrienes, and chemokines (Purohit et al. 2008);
- Activating signaling pathways that lead to proinflammatory cytokine release associated with liver fibrosis (Seki and Schnabl 2012); and
- Activating immune cells that can lead to liver inflammation and eventual fibrosis (Szabo et al. 2012).

## Gut–Brain Axis

It is well established that the brain helps control the gut, and recently research suggests the opposite also is true: the gut can influence brain function (Hsiao et al. 2013). In fact, some evidence suggests that alcohol-induced intestinal permeability and LPS can influence psychological and cognitive function. For example, among a group of alcohol-dependent, noncirrhotic patients hospitalized for detoxification, the subset that showed signs of intestinal permeability and LPS also had higher scores on measures of depression, anxiety, and alcohol cravings and scored worse on measures of selective attention (Leclercq et al. 2012). These findings suggest that some of the biological and behavioral changes seen in people with AUD may extend from the systemic inflammatory response triggered by changes in the gut.

Although the mechanisms by which the gut–brain axis conveys the effect(s) of alcohol on the central nervous system (CNS) are not well established, several studies suggest that systemic inflammation, like that caused by alcohol-provoked leaky gut, can influence the nervous system in several ways. For example, alcohol-induced gut inflammation can result in a systemic inflammation that subsequently affects neuronal function and may drive some symptoms of alcohol withdrawal, including autonomic disturbances and anxiety (Retson et al. 2015). In addition, elevated cytokines caused by the inflammatory response may be able to enter the brain and disrupt the blood–brain barrier, starting a vicious cycle that perpetuates alcohol’s effects on the CNS (Banks et al. 2015). Alcohol-induced dysbiosis may have its own effect on the CNS via vagal afferent nerve fibers, which influence areas of the brain implicated in AUD, including the thalamus, hippocampus, amygdala, and prefrontal cortex. Specifically, accumulating evidence suggests that alcohol-induced dysbiosis and gut microbiome may contribute to modifications in the vagal response and neuroinflammation in the CNS linked with alcohol-associated behaviors (Gorky et al. 2016). Other studies link microbiota alterations and endotoxins with neuroinflammation (Szabo and Lippai 2014) and anxiety-like behavior (Bercik et al. 2011; Hsiao et al. 2013). Studies in mice and humans suggest that antimicrobials and probiotics can positively influence brain function in healthy people, holding out promise that targeting gut microbiota in people with AUD might help defray alcohol’s influence on brain function (Bercik et al. 2011; Tillisch et al. 2013).

## Conclusions

Through multiple pathways, alcohol induces gut inflammation, which in turn promotes broad-spectrum pathologies both inside and outside the GI tract. In fact, many alcohol-related disorders, including cancers, liver disease, and neurological pathologies, may be exacerbated or directly affected by this alcohol-induced gut inflammation. The inflammation itself results from oxidative and nonoxidative pathways of alcohol metabolism that lead to a leaky gut, bacterial overgrowth, dysbiosis, and alterations in the mucosal immune system. As research uncovers the mechanisms by which alcohol affects gut inflammation and how that inflammation influences disease, researchers may be able to develop better strategies to prevent, or treat, conditions associated with chronic alcoholism. Already, studies are suggesting ways to modify diet and intestinal flora that may help alleviate some of the risks associated with chronic heavy drinking. Controlled trials are needed to assess the use of dietary supplementation with micronutrients in preventing or reversing alcohol effects.

## Figures and Tables

**Figure:**
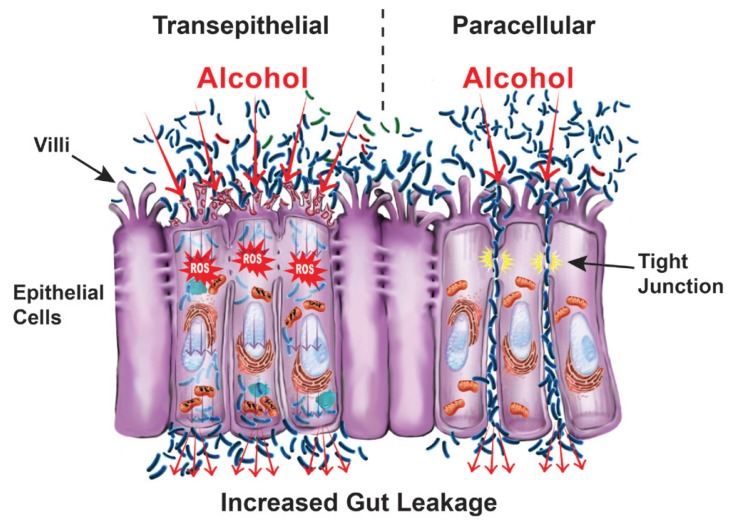
The intestinal barrier regulates the passage of materials, including microbial products, between the inside of the intestine (where food and drink go) and the cells and blood vessels on the other side of the epithelial cell layer lining the inside of the intestine. Alcohol disrupts the intestinal barrier, increasing its permeability, in two ways: via transepithelial mechanisms (cells on the left), which allow material to pass directly through the epithelial cells, and paracellular mechanisms (cells on the right), which allow material to pass through the junctions between the epithelial cells. Alcohol and its metabolites trigger transepithelial mechanisms by damaging the cells directly and weakening cell membranes via several mechanisms including oxidative stress caused by reactive oxygen species (ROS). Alcohol’s metabolites trigger paracellular mechanisms by disrupting the proteins that create the tight junctions linking cells and proteins that stabilize cells’ cytoskeletons. Increased permeability of the intestinal barrier allows bacteria and the toxins they create to leave the gut and infiltrate other organs through the bloodstream.
